# The spatial scale of dispersal revealed by admixture tracts

**DOI:** 10.1111/eva.12829

**Published:** 2019-06-27

**Authors:** Maud Duranton, François Bonhomme, Pierre‐Alexandre Gagnaire

**Affiliations:** ^1^ ISEM, Univ Montpellier, CNRS, EPHE, IRD Montpellier France

**Keywords:** admixture tracts, connectivity, dispersal, introgression, spatial genetics

## Abstract

Evaluating species dispersal across the landscape is essential to design appropriate management and conservation actions. However, technical difficulties often preclude direct measures of individual movement, while indirect genetic approaches rely on assumptions that sometimes limit their application. Here, we show that the temporal decay of admixture tracts lengths can be used to assess genetic connectivity within a population introgressed by foreign haplotypes. We present a proof‐of‐concept approach based on local ancestry inference in a high gene flow marine fish species, the European sea bass (*Dicentrarchus labrax*). Genetic admixture in the contact zone between Atlantic and Mediterranean sea bass lineages allows the introgression of Atlantic haplotype tracts within the Mediterranean Sea. Once introgressed, blocks of foreign ancestry are progressively eroded by recombination as they diffuse from the western to the eastern Mediterranean basin, providing a means to estimate dispersal. By comparing the length distributions of Atlantic tracts between two Mediterranean populations located at different distances from the contact zone, we estimated the average per‐generation dispersal distance within the Mediterranean lineage to less than 50 km. Using simulations, we showed that this approach is robust to a range of demographic histories and sample sizes. Our results thus support that the length of admixture tracts can be used together with a recombination clock to estimate genetic connectivity in species for which the neutral migration‐drift balance is not informative or simply does not exist.

## 1 INTRODUCTION

Demographic connectivity among populations plays an important role in the dynamics and resilience of species. First, it ensures the stability of local populations whose growth rate or persistence depends on immigration due to low local birth rates or high mortality rates (Furrer & Pasinelli, 2016; Pulliam, 1988; Runge, Runge, & Nichols, 2006). Demographic connectivity also contributes to the overall stability of metapopulations, by increasing the colonization potential of empty patches (Hanski, 1998). Therefore, improving empirical knowledge of species dispersal capabilities is of prime importance for understanding the ecoevolutionary dynamics of natural populations and provides helpful information for designing management and conservation actions (Lowe & Allendorf, 2010).

Quasi‐direct measures of individual movement between populations can be obtained using methods such as capture–mark–recapture field experiments, parentage analyses, or assignment tests. These approaches enable evaluating how net immigration contributes to population growth relative to local recruitment (Lowe, 2003), that is, demographic connectivity. However, they are often extremely difficult to implement (Broquet & Petit, 2009), especially for marine species in which dispersal usually takes place during a larval stage (Selkoe et al., 2016).

Indirect genetic approaches provide less demanding alternatives to evaluate average dispersal rates and distances (Broquet & Petit, 2009), although they remain uninformative regarding the contribution of dispersal to population demography, and hence stability (Lowe & Allendorf, 2010). Estimation of dispersal scales from isolation‐by‐distance (IBD) patterns (Rousset, 1997) has been used with success in several marine species (Palumbi, 2003; Pinsky, Montes Jr., & Palumbi, 2010; Pinsky et al., 2017; Puebla, Bermingham, & McMillan, 2012), sometimes providing consistent estimates of single‐generation dispersal distances compared to direct parentage assignment methods (Pinsky et al., 2017). Nevertheless, these methods are associated with a number of assumptions which potentially limit their range of application, such as equilibrium conditions between migration and drift. Equilibrium can take a long time to establish in species with large effective population sizes, which is commonly the case in marine species. Moreover, an independent assessment of the effective density of reproducing individuals (i.e., capturing drift effects) is required for estimating the standard deviation of dispersal distances from IBD patterns (Rousset, 1997). Therefore, such approaches are not always applicable even though IBD patterns are often observed in marine species (Selkoe et al., 2016).

The recent availability of genomewide polymorphism data in nonmodel organisms has opened new research avenues for assessing connectivity, especially with the information contained in selected and hitchhiker loci (Gagnaire et al., 2015). On the other hand, a renewed interest in neutral inferences has been shown thanks to the availability of haplotype data, which have a high potential to shed light on dispersal (Cayuela et al., 2018; Gagnaire et al., 2015; Pool & Nielsen, 2009). For instance, long identical‐by‐descent (IBD) blocks shared between individuals have been used to infer recent demography (Palamara & Pe'er, 2013; Ringbauer, Coop, & Barton, 2017). Similarly, the distribution of migrant tracts has also proved useful for inferring the timing of recent admixture events (Gravel, 2012; Pool & Nielsen, 2009). This second type of approach relies on the fact that gene flow between divergent gene pools (e.g., populations, lineages, subspecies, ecotypes) allows migrant chromosomes to enter a new genetic background with which they recombine. As migrant chromosomes diffuse through the landscape within the introgressed population, they are progressively shortened by recombination at each generation (Liang & Nielsen, 2014; Pool & Nielsen, 2009). Therefore, the length of migrant tracts (also called admixture tracts) is informative of the time elapsed since introgression, while being relatively robust to the effect of effective population size (Racimo, Sankararaman, Nielsen, & Huerta‐Sánchez, 2015). Analyzing the migrant tract length distribution in a spatial context should therefore enable to estimate the speed at which migrant tracts diffuse within an introgressed lineage and to ultimately estimate single‐generation dispersal distances on conservation‐relevant timescales. This method only requires gene flow between genetically differentiated populations and mapped SNPs to detect and measure the length of introgressed tracts using local ancestry inference (LAI). LAI methods have been developed that work even without the need to use phased markers (Baran et al., 2012; Guan, 2014). Nevertheless, a large variety of direct (Snyder, Adey, Kitzman, & Shendure, 2015) and indirect (Browning & Browning, 2011; Rhee et al., 2016) phasing methods can be used to facilitate the delineation of migrant tracts. Since admixture between diverging lineages is relatively common in nature (Payseur & Rieseberg, 2016), introgressed tracts have the potential to bring new information on dispersal in many species that remain difficult to study with direct tagging or classical indirect genetic approaches, which is the case for many marine species.

To illustrate this approach, we apply this framework to estimate the spatial scale of dispersal in a highly exploited marine fish, the European sea bass (*Dicentrarchus labrax*). This species is subdivided into an Atlantic and a Mediterranean glacial lineage, which started to diverge in allopatry about 300,000 years BP and remain currently partially reproductively isolated (Tine et al., 2014). Since the end of the last glacial period, asymmetrical gene flow allows the entry of Atlantic migrant tracts within the western Mediterranean population. In a recent study, we showed that these migrant tracts are on average shorter in the eastern compared to the western Mediterranean population, consistent with the action of recombination during the diffusion of Atlantic haplotypes across the Mediterranean Sea (Duranton et al., 2018). Here, we estimate the spatial scale of dispersal within the Mediterranean sea bass lineage by comparing the length distribution of introgressed Atlantic tracts between two different populations located at different distances from the contact zone with the Atlantic lineage. Furthermore, we use simulations to evaluate the robustness and the generality of this strategy to different admixture histories and sample sizes. With the development of new LAI methods to estimate the length distribution of admixture tracts (Corbett‐Detig & Nielsen, 2017; Medina, Thornlow, Nielsen, & Corbett‐Detig, 2018), we expect that quantitative assessments of dispersal will be obtained in several other species, which may help to resolve a long‐standing issue in conservation biology.

## 2 MATERIALS AND METHODS

### 2.1 Whole‐genome resequencing, phasing, and local ancestry inference

**Figure 1:**
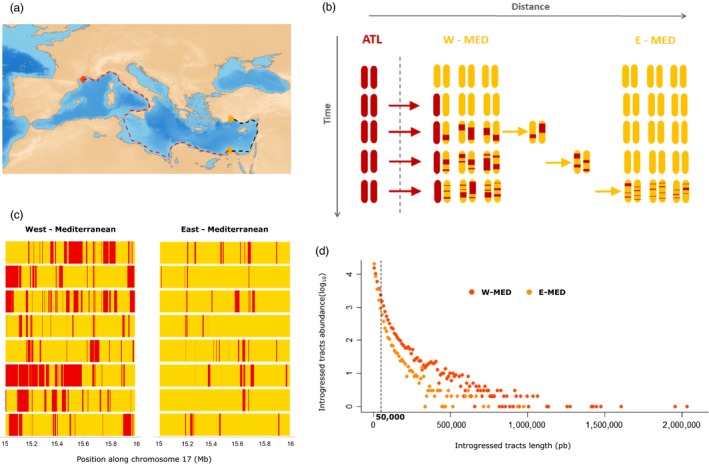
Introgression and diffusion of Atlantic tracts within the Mediterranean genetic background. (a) Geographical map showing the least cost dispersal distance of European sea bass (dotted lines) in continental waters of less than 200 m deep, between the western and the eastern Mediterranean Sea. Colored circles represent the geographical locations of western (orange) and eastern (yellow) Mediterranean samples. (b) Schematic representation of the diffusion‐recombination process of Atlantic introgressed tracts over time and space in the Mediterranean Sea. At each time step, 2 chromosomes are represented for the Atlantic population and 6 for the western and eastern Mediterranean populations. Blocks of Atlantic ancestry are colored in red, and Mediterranean tracts are in yellow. Red arrows represent the transfer of Atlantic blocks into the Mediterranean background through hybridization, and yellow arrows represent the diffusion of Atlantic blocks due to gene flow among populations within the Mediterranean Sea. (c) Schematic representation of the mosaic of ancestry tracts from the Atlantic (red) and Mediterranean (yellow) populations in a 1 Mb region of chromosome 17 for 8 haplomes from the western and eastern Mediterranean populations. (d) Genomewide distribution of introgressed tract length for the western (orange, W‐MED) and eastern (yellow, E‐MED) Mediterranean populations, using four individuals for each population. The vertical dotted line represents the threshold length value (50 kb) below which we do not consider introgressed tracts to estimate the difference in introgression times between sampling locations

Our analysis relies on the use of haplotype‐resolved whole‐genome sequences already published in Duranton et al. (2018). Briefly, we sequenced different mother–father–child trios obtained in experimental crossings to perform chromosome‐wide phasing‐by‐transmission (Browning & Browning, 2011). Females from the western Mediterranean Sea (Gulf of Lion, *n* = 8, ♀_W‐MED_) were crossed with males from either the Atlantic Ocean (English Channel, *n* = 4, ♂_ATL_) or the eastern Mediterranean Sea (Turkey *n* = 2 and Egypt *n* = 2, ♂_E‐MED_) to generate 8 families: 4 ♂_ATL_ × ♀_W‐MED_ and 4 ♂_E‐MED_ × ♀_W‐MED_ (Figure 1a). This sampling design allowed generating phased whole‐genome sequences from Mediterranean populations located at different distances from the contact zone with the Atlantic lineage (either near: W‐MED, or far: E‐MED). Since no genetic differentiation has been found between samples from Egypt and Turkey (Duranton et al., 2018), all E‐MED individuals were grouped together within a single population. Low‐quality and unphased genotypes were filtered to only retain sites with unambiguous transmission patterns and no missing data.

The filtered dataset consisting of 2,628,725 SNPs fully phased into chromosome‐wide haplotypes was used to perform LAI (Duranton et al., 2018). Blocks of Atlantic origin introgressed into the Mediterranean genetic background were identified along each individual chromosome haplotype with Chromopainter (Lawson, Hellenthal, Myers, & Falush, 2012). We then refined the delineation of tract junctions to generate the length distribution of Atlantic migrant tracts separately for the western and eastern Mediterranean populations. The limited sampling size for each population in our trio design was largely compensated by the amount of haplotype information per sample, since each individual genome is composed of a mosaic of hundreds of Atlantic and Mediterranean tracts. Therefore, only a small number of phased whole‐genome sequences provided sufficient information to obtain a clear picture of the admixture tract length distribution for the W‐MED and E‐MED populations (Duranton et al., 2018). This important aspect of the implemented methodology was also assessed using simulations (see below).

### 2.2 Estimation of introgression time from migrant tract length

Once introgressed within a divergent genetic background, migrant tracts are progressively shortened by recombination across generations (Liang & Nielsen, 2014; Pool & Nielsen, 2009). Therefore, long migrant tracts are expected to have introgressed on average more recently than short migrant tracts. In the European sea bass, blocks of Atlantic ancestry must enter the Mediterranean Sea from its western side near the Gibraltar strait before they diffuse eastward (Figure 1a). This diffusion across the Mediterranean seascape takes a certain number of generations during which migrant tracts are eroded by recombination (Figure 1b). Therefore, the shift between the migrant tracts length distributions of two sampling locations at different distances from the contact zone directly reflects the time it takes for an Atlantic haplotype to diffuse from the nearest to the farthest location with respect to the contact zone. Here, we estimated the time of entrance for Atlantic tracts found in western and eastern Mediterranean populations and calculated the difference between these two estimates to evaluate the average time for a migrant tract to cross the Mediterranean Sea. To estimate the time of entrance in each location, we focused on neutral genomic regions and used an analytical expectation for the mean length of migrant tracts ($\overset{¯}{L}$) as a function of the time since initial admixture (*t*, expressed in generations), the admixture proportion of the population considered (*f*, the fraction of Atlantic ancestry), and the local recombination rate (*r*, in Morgan per base pair) (Racimo et al., 2015).

(1) $$\overset{¯}{L}={\left[\left(1-f\right)r\left(t-1\right)\right]}^{-1}$$

### 2.3 Data filtering

The length distribution of migrant tracts is influenced by the temporal dynamics of introgression. Given that gene flow has been introducing Atlantic alleles within the Mediterranean since the end of the last glacial period (Tine et al., 2014), haplotypes of variable ages (and therefore variable lengths) are expected to be found at any Mediterranean location. The shortest tracts that reside in the Mediterranean for a much longer time than it takes to diffuse from west to east have very similar lengths among locations. Therefore, the shift in the length of Atlantic tracts between western and eastern Mediterranean population is all the more important that the tracts have introgressed recently and therefore remain long (Figure 1c,d). For that reason, we only considered blocks of Atlantic ancestry longer than 50 kb, since shorter blocks are less informative for estimating recent introgression. Applying a length threshold to remove short tracts has been also used to control for technical limitations to measure short introgressed tracts (Ni et al., 2016). Moreover, because migrant tracts length is more difficult to estimate in highly recombining regions of the genome, we excluded such regions from the analysis. In the sea bass genome, local recombination rates tend to be markedly reduced in central chromosomal regions (*ρ* = 4*N*_e_r usually <5 per kb) compared to chromosome extremities (*ρ* usually >40 per kb) (Tine et al., 2014). We thus applied a population‐scaled recombination rate threshold of *ρ* = 10 per kb to keep only low‐recombining regions.

Our analysis of migrant tract length relies on a neutral theory. In order to avoid potentially confounding effects of selection against Atlantic alleles, we filtered genomic regions that probably contain barrier loci that locally reduce gene flow between Atlantic and Mediterranean lineages. These regions, which represent ~4% of the genome (Duranton et al., 2018), were identified using the *RND*_min_ statistics (Rosenzweig, Pease, Besansky, & Hahn, 2016) calculated in 100 kb windows. This minimum relative node depth statistics corresponds to the ratio of the minimal value of *d*_XY_ calculated between all Atlantic and eastern Mediterranean individuals (*d*_min_) to the mean value of *d*_XY_ measured between *D. labrax* and the outgroup species *Morone saxatilis* (*d*_out_). An empirical upper threshold *RND*_min_ value of 0.03 (i.e., corresponding to the 95th percentile of the distribution) was used to conservatively exclude genomic regions influenced by selection from our dataset, according to previous results (Duranton et al., 2018).

### 2.4 Estimation of the average tract length ($\overset{¯}{L}$)

We used two different approaches to estimate the average introgressed tract length for each of the two Mediterranean populations. Our first method specifically addresses the direct influence of local recombination rate (*r*) variations on the length distribution of introgressed tracts. Broadscale variation in recombination rate along chromosomes is commonly observed in eukaryotes (Haenel, Laurentino, Roesti, & Berner, 2018), including teleost fishes (Bradley et al., 2011; Roesti, Hendry, Salzburger, & Berner, 2012) and among them the European sea bass (Tine et al., 2014). As a result, the length of migrant tracts is expected to decrease at variable rates across the sea bass genome, even though we excluded the most highly recombining regions from our analysis. To account for these variations, we calculated the average length of introgressed tracts locally within nonoverlapping 100 kb windows, using all introgressed tracts that were either fully contained within, or simply overlapping each focal window. The average time since introgression was then estimated separately for each window using equation (1) with the average length of introgressed tracts and the local recombination rate value estimated for that window (Tine et al., 2014). We also used the observed genomewide admixture proportions for the western (*f* = 0.31) and eastern (*f* = 0.13) Mediterranean populations (Duranton et al., 2018). After removing windows showing no introgressed tracts, we retained a total of 2,092 and 1,065 windows for the western and eastern Mediterranean populations, respectively. We then merged time estimates across windows to generate the distribution of introgression times separately in each Mediterranean population. Finally, we bootstrapped both distributions 10,000 times and identified the maximum value of each bootstrap replicate. We then used the 0.025 and 0.975 quantile values of bootstrapped maxima to estimate a 95% confidence interval for the maximum value of the distribution in each population. The difference between the two maxima represents the average number of generations taken for a migrant tract to cross the Mediterranean Sea.

The second method builds on the fact that the abundance of introgressed tracts as a function of their length follows an exponential distribution (Gravel, 2012; Pool & Nielsen, 2009; Racimo et al., 2015) with a mean $\overset{¯}{L}=\frac{1}{\lambda }$, where *λ* corresponds to the rate parameter of the exponential distribution. To estimate *λ*, we log‐transformed the tract abundance values from the introgressed tract length distribution to obtain a linear distribution which slope equals −*λ*. In order to estimate this slope using data from similar recombination rate regions, genomic windows of 100 kb were grouped into eleven recombination rate categories, which were designed to receive an equal number of windows (i.e., 209 windows in each category). For each category, we then separated the tracts into twenty bins of tract length and used only bins with at least five tracts to fit the linear regression. Finally, we plotted the values of $\lambda =\frac{1}{\;\overset{¯}{L}}$ estimated for every recombination rate category as a function of the average recombination rate of the 209 windows used in the corresponding category. We fitted a linear regression to this distribution forcing the intercept to equal zero and determined its slope *a* = (1 − *f*)(*t* − 1), (where *f* corresponds to the admixture proportion) which allowed us to estimate the time since introgression as $t=\frac{a}{1-f}+1$ separately for the eastern and western Mediterranean populations. The difference between the two estimates corresponds to the number of generations necessary for a track to diffuse from west to east.

### 2.5 Estimate of the least coast distance between the Mediterranean populations

We used the R package marmap (Pante & Simon‐Bouhet, 2013) to estimate the least cost distances between western and eastern Mediterranean sampling locations. Since the European sea bass is a neritic benthopelagic species occupying shallow continental waters (Pickett & Pawson, 1994), we considered that dispersal only occurs through areas where the maximum depth is less than 200 m. We estimated the distance between the western and both north and southeastern Mediterranean locations as *Dist*_west‐south_east_ = 4,891 km and *Dist*_west‐north_east_ = 6,005 km (Figure 1a). Since we analyzed all eastern individuals together without separating northern from southern samples, we used the average distance *Dist*_west‐east_ = 5,448 km between the western and the two eastern populations to calculate the per‐generation dispersal distance.

### 2.6 Validation of the methodology by simulations

We used neutral simulations to test whether the length distribution of introgressed tracts can be used to reliably estimate the diffusion time of Atlantic haplotypes between western and eastern Mediterranean populations. To do so, we used the coalescent simulator msprime v0.6.2 (Kelleher, Etheridge, & McVean, 2016) to simulate the length distribution of Atlantic tracts introgressed within the western Mediterranean population under a secondary contact model (see legend of Figure 4a). Demographic and temporal simulation parameters were set to values that were previously shown to accurately reproduce this distribution (Duranton et al., 2018). We then aimed at modeling the diffusion of introgressed tracts from the western toward the eastern Mediterranean population using the same simulation framework. This diffusion is characterized by the decay of introgressed haplotype length due to the recombination events that occur every generation during the time it takes to cross the Mediterranean Sea, and it is therefore not directly influenced by the Atlantic population. To model this diffusion, we thus considered a third episode lasting for *T*_diff_ generations, in which gene flow between the Atlantic and western Mediterranean population stops. During these *T*_diff_ generations, the erosion of introgressed haplotypes that are not renewed anymore by gene flow from the Atlantic mimics what happens when haplotypes diffuse away from the western to reach the eastern Mediterranean population. In this way, the spatial diffusion process of introgressed tracts is simply modeled by reproducing the temporal erosion of introgressed tracts after they enter the western Mediterranean, without making spatially explicit simulations. To identify and measure the exact length of introgressed tracts within simulated Mediterranean genomes, we used functions implemented in Skov et al., (2018), which track the local ancestry of recombining genomic segments during the course of the coalescent simulations. We simulated a single chromosome of 25 Mb (i.e., the average length of *D. labrax* chromosomes) with constant recombination and mutation rates of 6.84e^−8^ (i.e., the mean recombination rate calculated across the sea bass genome) and 1e^−8^, respectively.

To estimate the introgression time from simulated tracts sampled at times Date_*T*_diff_ and Date*_T*_0_ within the Mediterranean population, we used Equation (1) in which $\overset{¯}{L}$ was calculated as the average length of introgressed tracts and *f* as the genomic fraction occupied by introgressed tracts. The difference between the estimated time since introgression at Date*_T*_0_ and Date_*T*_diff_ represents the diffusion time of Atlantic haplotypes (i.e., parameter *T*_diff_ in our model), as they move from the western to the eastern Mediterranean population.

Our first objective was to determine whether the proposed approach allows to reliably estimate the diffusion time parameter *T*_diff_. We therefore parameterized our historical demographic model using the same parameter values as in Duranton et al., (2018) (i.e., *T*_div_ = 54,000 generations, *T*_c_ = 2,300 generations and *N*_MED_*m_1_ = 7 migrants per generation) and explored a range of *T*_diff_ values spanning those estimated between the two Mediterranean populations (i.e., 20, 50, 150, 350, 550, 800, 1,200, and 1,600 generations). We performed 10 replicate simulations for each *T*_diff_ value and sampled 4 individuals per population at each sampling time, which corresponds to the number of individuals used in this study.

Our second objective was to determine the robustness of our method to different sample sizes and demographic histories. In order to assess the effect of the amount of data, we used the parameter values of the European sea bass (i.e., *T*_c_ = 2,300 and *T*_diff_ = 550 generations, *N*_MED_*m_1_ = 7 migrants per generation) and sampled either 1, 2, 3, 4, 5, 6, or 7 individuals at each time point. Simulations were run 10 times for each value, and *T*_diff_ was estimated for every replicate. We then fixed the number of sampled individuals to 4 and made the secondary contact duration parameter (*T*_c_) vary around the estimated number of generations of admixture in the sea bass (i.e., using *T*_c_ = 250, 500, 1,000, 1,500, 2,300, 3,500, and 5,000). We then did the same for the number of migrants entering the Mediterranean population per generation (using *N*_MED_*m_1_ = 1, 3, 5, 7, 10, 15, and 20). These explored values allowed us to consider a wide range of gene flow durations and intensities, which partly covers the diversity of settings found in other species. We did not make the divergence time (*T*_div_) vary since this parameter mostly influences the accuracy of the detection of introgressed tracts, which were called directly in our simulations instead of being inferred with a LAI method as we did from real data.

## 3 RESULTS

### 3.1 Dating introgression in nonoverlapping 100 kb windows

**Figure 2:**
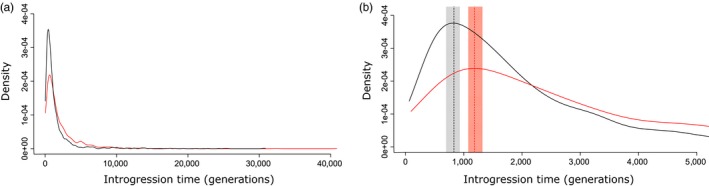
Distributions of estimated time since introgression. Times were estimated in non‐overlapping 100 kb windows, separately for the western (black) and eastern (red) Mediterranean populations. (b) Dotted vertical bars show the maximum of each distribution, and shaded rectangles represent their 95 percent confidence intervals

Our first method, which estimates the time since introgression using the average length of admixture tracts in nonoverlapping 100 kb window, specifically accounted for local recombination rate variation among windows. By combining the estimated times across all retained windows, we obtained a distribution of introgression time for the western and eastern Mediterranean populations, separately (Figure 2). The two distributions showed similar shapes (Figure 2a), except that more recently introgressed Atlantic tracts were observed in the western compared to the eastern Mediterranean population. Furthermore, the eastern distribution was slightly shifted toward longer introgression times, indicating that introgressed tracts are on average older in this population (Figure 2b). We estimated the maximum of each distribution and its 95 percent confidence interval as *T*_west_max_ = 831.01 (CI95% = [721.55; 967.81]) and *t*_east_max_ = 1,186.15 (CI95% = [1,071.48; 1,329.47]) generations for the western and eastern Mediterranean, respectively. Therefore, there was a difference in diffusion time of *t*_diff_1_ = 1,186.15–831.01 = 355.14 (CI95% = [103.67; 607.92]) generations between locations. This difference corresponds to a per‐generation dispersal distance of *d*_west‐east_1_ = 5,448/355.14 = 15.34 km (CI_95%_ = [8.96; 52.55]).

### 3.2 Dating introgression using the log‐transformed distribution of tracts length

**Figure 3:**
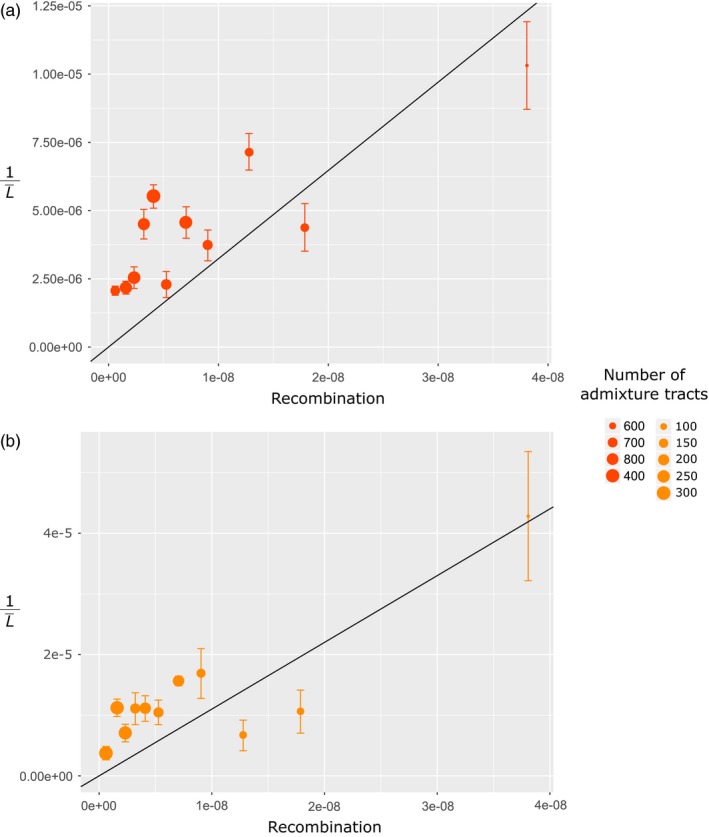
Correlations between $\frac{1}{\;\overset{¯}{L}}$ and the average recombination rate estimated for each of the eleven recombination rate categories (each containing 209 genomic windows) for the eastern (a) and western (b) Mediterranean population. The size of the points indicates the number of admixture tracts contained in the 209 windows of each recombination rate category, and the vertical bars indicate the standard error of the estimated statistics. The black line shows the linear regression fitted for each population

Our second method that modeled the log‐transformed distribution of admixture tracts length to estimate the mean tract length ($\overset{¯}{L}$) relied on the analysis of 2,299 windows grouped into eleven recombination rate categories (Table 1). Although we delimited the range of each recombination rate category to evenly distribute windows across categories, the total amount of information slightly differed among categories due to varying amounts of admixture tracts per window. For that reason, the slope of the regression of the log‐transformed distribution of admixture tracts length was only marginally significant for some recombination rate categories with limited amount of data (i.e., five categories in the eastern population, Table 1). As expected, the estimated average length of introgressed tracts was shorter in the eastern compared to the western population for every category. We then plotted the eleven estimated values of $\frac{1}{\;\overset{¯}{L}}$ as a function of their corresponding recombination rate, separately for the western and eastern Mediterranean populations (Figure 3). We estimated the slope of the linear regression to *a*_west_ = 323.49 (*SE* = 53.23; *R*‐squared = 0.77 and *p*‐value = 1.19e^−4^) for the western and to *a*_east_ = 1,100.2 (*SE* = 151.67; *R*‐squared = 0.82 and *p*‐value = 2.75e^−5^) for the eastern population. Using these values, we estimated *t*_west _to 469.83 (CI95% = [315.54; 624.12]) and *t*_east_ to 1,265.60 (CI95% = [916.92; 1,265.60]) generations. Thus, we estimated a diffusion time *t*_diff_2_ = 1,265.60–469.83 = 795.77 generations (CI95% = [292.8; 950.06]) and a per‐generation dispersal distance of *d*_west‐east_2_ = 5,448/795.77 = 6.85 km per generation (CI95% = [5.73; 18.61]).

**Table 1:** Summary statistics of the linear correlations between the log‐transformed number of admixture tracts and their length in eleven categories of local recombination rate

Recombination rate (M per bp)	Number of windows	Population	Number of tracts	$\lambda =\frac{1}{\;\overset{¯}{L}}$	*SE*	*R*‐squared	*p*‐value
6.10e^−10^	209	W‐MED	689	2.06e^−6^	1.66e^−7^	0.93	**2.14e^−7^**
		E‐MED	306	3.74e^−6^	1.10e^−6^	0.57	0.012
1.59e^−9^	209	W‐MED	831	2.17e^−6^	2.35e^−7^	0.86	**4.38e^−7^**
		E‐MED	294	1.12e^−5^	1.44e^−6^	0.90	**5.55e^−4^**
2.32e^−9^	209	W‐MED	856	2.54e^−6^	3.95e^−7^	0.76	**3.21e^−7^**
		E‐MED	283	7.06e^−6^	1.46e^−6^	0.67	**6.73e^−4^**
3.21e^−9^	209	W‐MED	823	4.50e^−6^	5.42e^−7^	0.88	**3.32e^−5^**
		E‐MED	174	1.11e^−5^	2.63e^−6^	0.67	**0.004**
4.09e^−9^	209	W‐MED	931	5.52e^−6^	4.27e^−7^	0.95	**3.88e^−6^**
		E‐MED	219	1.11e^−5^	2.11e^−6^	0.75	**7.75e^−4^**
5.26e^−9^	209	W‐MED	736	2.29e^−6^	4.77e^−7^	0.65	**5.52e^−4^**
		E‐MED	175	1.05e^−5^	2.03e^−6^	0.74	**8.63e^−4^**
7.064e^−9^	209	W‐MED	874	4.56e^−6^	5.76e^−7^	0.86	**2.41e^−5^**
		E‐MED	218	1.56e^−5^	7.81e^−7^	0.99	**2.72e^−4^**
9.040e^−9^	209	W‐MED	713	3.72e^−6^	5.65e^−7^	0.71	**6.26e^−6^**
		E‐MED	144	1.69e^−5^	4.10e^−6^	0.76	0.014
1.278e^−8^	209	W‐MED	657	7.15e^−6^	6.70e^−7^	0.90	**3.80e^−7^**
		E‐MED	116	6.66e^−6^	2.52e^−6^	0.40	0.029
1.786e^−8^	209	W‐MED	652	4.38e^−6^	8.73e^−7^	0.67	**3.90e^−4^**
		E‐MED	112	1.06e^−5^	3.55e^−6^	0.57	0.031
3.807e^−8^	209	W‐MED	556	1.03e^−5^	1.60e^−6^	0.83	**3.57e^−4^**
		E‐MED	76	4.28e^−5^	1.06e^−5^	0.83	0.057

Note

Model summaries are presented separately for the western and eastern Mediterranean populations. The *p*‐values of significant regressions after applying Bonferroni correction for multiple tests (*p* < 0.0045) appear in bold.

### 3.3 Validation of the methodology using simulations

**Figure 4:**
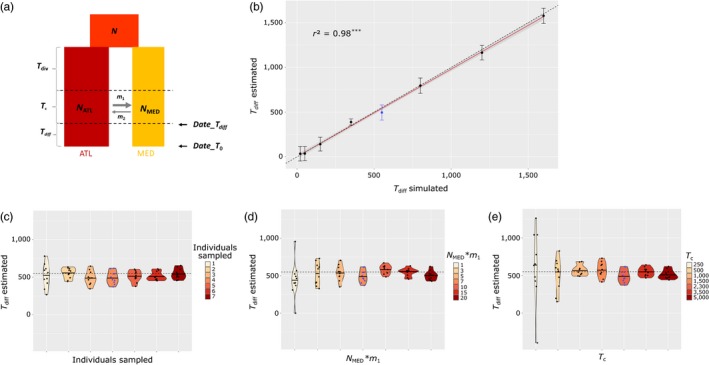
Reliability evaluation of the approach to estimate the diffusion time separating two sampling time points. (a) Schematic representation of the model used in simulations. An ancestral population of size N splits into two populations ATL and MED of size N_ATL_ and N_MED_, which diverge during *T*_div_ generations and then exchange genes for *T*_c_ generations and then follows a third episode without gene flow lasting for *T*_diff_ generations, which represents the diffusion period during which the length of introgressed tracts is only influenced by the erosion process in the recipient population. The Mediterranean population is sampled two times, the first one at the beginning of the period without gene flow (Date_*T*_diff_) and the second one at the end (date_*T*_0_). The migration rates from the ATL to the MED population and in the opposite direction are represented by parameters m_1_ and m_2_, respectively. (b) Correlation between the estimated and simulated values of *T*_diff_ using 4 individuals per sampling time point, *T*_c_ = 2,300 and m_1_*N_MED_ = 7. For each value of *T*_diff_, simulations were run 10 times, each point represents the average estimated value over replicates and the vertical bar the standard error. The dashed line represents the equation y = x, and the red line is the linear regression with its confidence interval in grey shade. Estimated values of *T*_diff_ using model parameters estimated for the European sea bass: *T*_c_ = 2,300, m_1_*N_MED_ = 7 and *T*_diff_ = 550 (represented by the dashed line) using (c) different sample sizes, (d) N_MED_*m_1_ values, and (e) durations of contact (T_C_). Points represent estimated *T*_diff_ values of the 10 replicate simulations for each tested value, and horizontal bars their median value. Blue points represent simulations that were modeled using the same model parameter values as in the European sea bass

We used simulated data to test whether the average length of introgressed tracts measured at two time points after entering a recipient population can be used to reliably estimate their diffusion time. We showed that there is a strong correlation between the simulated and estimated values of diffusion times (*T*_diff_) (Figure 4b). This indicates that measuring the difference in time since admixture between two populations connected by gene flow allows to accurately estimate the number of generations that it takes to connect them through dispersal. Although we did not explicitly consider the spatial diffusion process here, this should not strongly affect the realism of our simulations since the erosion of tracts mostly depends on recombination and time. Indeed, for neutral regions, the length distribution of introgressed tracts is not influenced by the effective population size. Furthermore, the equation used here to estimate the time since admixture assumes a unique pulse of historical admixture (Gravel, 2012; Racimo et al., 2015). Because we simulated a continuous period of gene flow, our results also indicate that the used formula remains applicable in a context of continuous migration.

We further tested if our estimations of diffusion time were robust to a range of sample sizes and demographic histories. Results were relatively stable across a wide range of sample sizes, even when only one individual was used (Figure 4c). To account for varying demographic histories, we modeled different durations and intensities of gene flow and showed that none of these parameters strongly impacts the estimation of diffusion time (Figure 4d and e). The variance in estimated values among replicates was high only in cases of low introgression levels, which were either due to a low migration rate or a short period of gene flow. This indicates that in such cases, introgressed tracts were not abundant enough to precisely estimate their average length with only four individuals sampled at each time point. Increasing the sample size in cases of limited introgression would thus probably improve the reliability of time estimates. It is also important to consider that we only simulated a single 25 Mb chromosome per replicate run due to computational limitations. However, real datasets such as the one used here in the European sea bass cover the whole genome (i.e., 24 chromosomes) and therefore contain larger amounts of data. Therefore, the proposed methodology is likely to give consistent results even with only small numbers of genomes sequenced in each population.

## 4 DISCUSSION

We used the information contained in the length of admixture tracts as a means to estimate the spatial scale of dispersal within a population receiving genetic material from a distinct lineage. Introgressed tracts entering a recipient population are progressively shortened every generation by recombination, providing a clock that keeps track of the history of introgression (Liang & Nielsen, 2014; Pool & Nielsen, 2009). Here, the proposed methodology relies on the fact that introgressed tracts get on average shorter when they reach locations farther away from the contact zone, a process considered to be relatively independent from the effective population size (Gravel, 2012; Racimo et al., 2015). The difference in tract length between two locations at different distances from the contact zone thus represents the action of recombination during the time needed to connect these two locations through multigenerational dispersal (Figure 1b) (Duranton et al., 2018). Using empirical data and coalescent simulations, we show that this approach provides a means to estimate the spatial scale of dispersal in populations that are not at migration–drift equilibrium.

Despite our efforts to filter the European sea bass data prior to the estimation of time parameters, a few confounding factors may have had undesirable effects on our analyses. The first one is selection against introgressed Atlantic tracts in some regions of the Mediterranean genomes. To control for that effect, we conservatively removed genomic windows that showed signs of selection against introgression, in order to consider only regions where Atlantic tracts have likely introgressed neutrally. A second possible source of bias comes from technical limitations of LAI methods to estimate the length of short introgressed tracts (Ni et al., 2016). This category of tracts is, however, less informative for the type of analysis presented here, since their whole history of recombination within the Mediterranean is likely much longer than the time needed to diffuse across the Mediterranean Sea. On the contrary, long migrant tracts are more likely to display contrasted lengths between remote locations. For these reasons, we did not consider highly recombining genomic windows (4*N*_e_r > 10) where the differential in tract length is rapidly lost, as well as introgressed fragments shorter than 50 kb in the remaining windows.

Although removing short tracts allows extracting the most informative fraction of the data, it may have biased the estimation of the time since introgression obtained from our first methodology. This filtering step should indeed slightly increase the average tract length within windows, which in turn should affect the distribution of the time since introgression. Therefore, the estimated time may be underestimated for both populations, but possibly more so for the eastern population that contains a relatively higher fraction of short tracts. Our filtering of short tracts may thus lead to an underestimation of the diffusion time between locations, that is, an overestimation of the per‐generation dispersal distance.

To overcome this potential difficulty, we used a second methodology that models the mean length of introgressed tracts from their log‐transformed distribution. As such, this approach is largely independent of the distribution tail and thus insensitive to removing short tracts. This filtering step only reduces the amount of data and therefore the power of the regression approach, but without modifying the regression slope. On the downside, this method needs to group windows with similar recombination rate values so that each category has enough introgressed tracts to perform powerful regressions. The average recombination rate value used for each of the eleven categories may cause some loss of precision regarding fine‐scale recombination rate variation, as compared to our first approach. However, windows within a given category displayed a small variance in recombination rate. Therefore, we speculate that averaging recombination rate values among windows within categories did not strongly affect our inferences. A result supporting this conjecture was the reasonably high amounts of variance in mean tracts length explained by the linear regression models fitted for each recombination rate category (Table 1).

Overall, our two methodologies can be seen as complementary. The first one allows to consider more fine‐scaled variations in recombination rate along the genome but might be sensitive to the removal of short tracts in the presence of historical admixture. The second approach is probably robust to the removal of short tracts but at the expense of summarizing variation in recombination rate within bins. Despite these differences, the two values of the time since introgression estimated for the eastern population (*t*_east_max_ = 1,186.15 (CI_95%_ = [1,071.48; 1,329.47]) and *t*_east_ = 1,265.60 (CI95% = [916.92; 1,265.60])) were very similar, and the second method provided an older estimate, as expected. The two values estimated for the western population (*T*_west_max_ = 831.01 (CI95% = [721.55; 967.81]) and *t*_west_ = 469.83 (CI95% = [315.54; 624.12])) were, however, more different, with a younger estimate obtained with the second method, which is contrary to our expectation. A possible explanation could be that since the western population is closer from the contact zone, it has a higher variance in tract length that reduces the precision of estimated time. Nevertheless, our two quantitative estimates of the per‐generation dispersal distance were very close to each other and displayed largely overlapping confidence intervals (first method: *d*_west‐east_1_ = 15.34 km (CI_95%_ = [8.96; 52.55]) and second method: *d*_west‐east_2_ = 6.85 km (CI95% = 5.73; 18.61])). As expected, the second method which is less prone to overestimate dispersal due to the removal of short tracts provided a smaller estimate the per‐generation dispersal distance. Since our simulation study also supported the validity of the implemented approach (Figure 4b), we are confident that our empirical numerical estimates provide reliable indications of the spatial scale of dispersal in the European sea bass.

One possible limitation of this study could be that the analytical expectation we used assumes a unique pulse of admixture (Gravel, 2012; Racimo et al., 2015), while gene flow between the two sea bass lineages has been ongoing since the last glacial retreat (Tine et al., 2014). Methods accounting for continuous gene flow (Gravel, 2012; Ni et al., 2016) provide more realistic modeling of the migration history but are more suitable for inferring recent admixture (i.e., around 100 generations). Therefore, such methods would have little power with cases of postglacial gene flow, as it is the case in the European sea bass. Furthermore, our simulations showed that using the theoretical expectation for a single pulse of admixture provides rather accurate results (Figure 4b). Therefore, our choice of methodology offers a good compromise considering the methods currently available. We anticipate, however, that the increasing accessibility to local ancestry information in population genomic studies will foster the development of new methods that better account for continuous historical gene flow.

Our numerical estimations seem consistent with previous population genetics studies demonstrating the existence of a genetic structure between western and eastern Mediterranean populations using allozymes (Allegrucci, Fortunato, & Sbordoni, 1997), microsatellite markers (Bahri‐Sfar, Lemaire, Hassine, & Bonhomme, 2000; Quéré et al., 2012), and SNPs (Souche et al., 2015). These results are also in line with the suspected philopatric behavior of the European sea bass (Bahri‐Sfar et al., 2000; Castilho & Ciftci, 2005; de Pontual et al., 2019). Nevertheless, since we only used two sampling locations for this proof‐of‐concept study, our estimated dispersal distances should be interpreted with caution. Indeed, they represent an average dispersal distance calculated between two distant populations. Therefore, inferred distances may be decreased by local dispersal barriers that reduce gene flow somewhere in between the two sampling sites. For instance, water circulation features in the Siculo‐Tunisian strait are known to reduce gene flow between the western and eastern Mediterranean populations in several Mediterranean fish species (but see Pascual, Rives, Schunter, & Macpherson, 2017) including sea bass (Bahri‐Sfar et al., 2000; Quéré et al., 2012). A finer‐scale sampling of the Mediterranean Sea could thus allow refining local estimates of dispersal distance to test and quantify the effect of such barriers on dispersal. Nonetheless, our estimates already provide relevant ecological information on the spatial scale of dispersal in Mediterranean sea bass for conservation and management purposes. Even accounting for uncertainty, our results indicate relatively short effective dispersal distances (<50 km per generation) considering the potential offered by the larval phase of 8 to 12 weeks and the mobility of juvenile and adult stages (Bahri‐Sfar et al., 2000). This illustrates the complex relationships existing between pelagic larval duration and gene flow in marine species (Nanninga & Manica, 2018; Selkoe & Toonen, 2011) and raises the question of the long‐distance benefits of marine reserves in terms of demographic connectivity (Manel et al., 2019).

Here, we used the European sea bass as a case study to illustrate the potential of admixture tracts for estimating dispersal in nonequilibrium populations. However, our main message is that similar approaches could be applied to a wide range of species, especially marine organisms in which estimating dispersal distances remains a challenging issue (Gagnaire et al., 2015). Despite the apparent lack of strong physical barriers to dispersal in the marine realm, genetic studies have shown that many species are subdivided into genetically interacting cryptic lineages, ecotypes, or partially reproductively isolated populations (see Bierne, Welch, Loire, Bonhomme, & David, 2011 for a review). For instance, even at the regional scale of the North Atlantic Ocean, such subdivisions have been illustrated by a number of studies in several commercial fish species including the Atlantic herring (*Clupea harengus*) (Guo, Li, & Merilä, 2016; Lamichhaney et al., 2012; Limborg et al., 2012; Martinez Barrio et al., 2016), the Atlantic cod (*Gadus morhua*) (Berg et al., 2016; Bradbury et al., 2014; Hemmer‐Hansen et al., 2013; Karlsen et al., 2013; Sodeland et al., 2016), the turbot (*Scophthalmus maximus*) (Nielsen, Nielsen, Meldrup, & Hansen, 2004; Vandamme et al., 2014), the European hake (*Merluccius merluccius*) (Milano et al., 2014; Nielsen et al., 2012), and the European anchovy (*Engraulis encrasicolus*) (Le Moan, Gagnaire, & Bonhomme, 2016). The lack of reliable measures of dispersal within and among populations of such species can generate mismatches between the delineation of fisheries management units and conservation objectives (Reiss, Hoarau, Dickey‐Collas, & Wolff, 2009). Moreover, spatial patterns of admixture can be confounded with evidence for either strong isolation‐by‐distance or local adaptation, since introgression gradients tend to increase genetic differentiation between populations located at different distances from a contact zone (Gagnaire et al., 2015). If not accounted for, such gradients may lead to an underestimation of dispersal distances from isolation‐by‐distance patterns. Furthermore, when introgression and environmental gradients are spatially overlapped, as it is commonly the case, it is very difficult to distinguish loci under local selection from introgression clines at neutral loci. Therefore, the risk of misinterpreting dispersal and local adaption patterns is particularly high when admixture occurs between genetically differentiated groups. A careful analysis of genetic variation including demographic inferences to reconstruct the evolutionary history of the studied populations thus appears to be a first important step in genetic connectivity studies.

An important prerequisite for applying the methodology developed in our study is to accurately identify and measure introgressed tracts. Although this may require phased haplotype data to perform LAI, haplotype phasing approaches are making this task increasingly feasible (Browning & Browning, 2011; Rhee et al., 2016). Having access to a chromosome‐level reference genome assembly will no longer remain necessary with haplotype‐resolved genome sequencing methods based on long read sequencing technologies (Browning & Browning, 2011; Snyder et al., 2015). In parallel to ongoing progress in sequencing, a wide variety of methods for LAI have been developed (Geza et al., 2018; Yuan et al., 2017). Some of them allow to correct phasing errors while identifying admixture tracts (Dias‐Alves, Mairal, & Blum, 2018; Maples, Gravel, Kenny, & Bustamante, 2013; Salter‐Townshend & Myers, 2018), making LAI accessible with data that are phased with a large variety of methods, including statistical phasing. Phased sequence data are even not necessary with some methods that only require the physical (Baran et al., 2012) or genetic (Guan, 2014) position of variants to identify ancestry blocks (Baran et al., 2012; Guan, 2014). Recently, a new method has also been developed to perform LAI directly from reads pileup data in population samples with arbitrary ploidy (Corbett‐Detig & Nielsen, 2017). Therefore, there is a good potential for using the length of tracts to date admixture, including with reduced‐representation sequencing data that still represent the most common type of data used in population genomic studies. An example of this kind of approach has been successfully applied with ddRAD SNPs for identifying introgressed tracts in supplemented populations of wild brown trout (Leitwein, Gagnaire, Desmarais, Berrebi, & Guinand, 2018).

The ability to correctly estimate the length of introgressed tracts admittedly depends on the density of markers. However, the minimal marker density required depends on the average length of introgressed tracts and therefore on the time since the beginning of admixture. The more recent the introgression is, the less the density of genetic markers is necessary. In all cases, the precise delineation of migrant tracts will be facilitated by a stronger divergence between admixing lineages (Gravel, 2012). Therefore, reduced‐representation sequencing approaches such as RAD‐sequencing, which can generate from 10 to 1,000 loci per Mb (Andrews, Good, Miller, Luikart, & Hohenlohe, 2016), can offer the flexibility suitable to date both recent admixture between young lineages and more ancient introgression between divergent lineages.

Finally, our simulation study showed that the proposed methodology can give accurate results for a wide range of sample sizes and demographic histories, and this for inferring a large range of diffusion times. For instance, assuming a demographic history similar to that of the European sea bass, we showed that a single individual may be sufficient to provide reliable results. More generally, the method can be applied either with a small number of whole‐genome sequences for cases of historical gene flow or using larger sample sizes with reduced‐representation sequencing data for studying recent admixture. Finally, because the method seems to be accurate over a large range of gene flow intensities and diffusion times, it can be used to measure dispersal at a more refined spatial scale than the one considered here.

## 5 CONCLUSION

Our study illustrates the potential of admixture tracts for estimating the spatial scale of dispersal in nonequilibrium populations, which is an essential parameter to design appropriate management and conservation actions. Although methodological improvements will be needed to better account for ancient migrations, the proposed approach provides a roadmap to generate valuable information on a conservation‐relevant timescale and is already well suited for species with relatively recent admixture histories. The development of new methods that simultaneously estimate local ancestry and the time since admixture (Corbett‐Detig & Nielsen, 2017) should further accelerate the interest for this kind of approach. This is especially true for species in which direct measures of dispersal are not applicable and the neutral migration–drift balance is not informative or simply does not exist, which is the case for many marine species.

## CONFLICT OF INTEREST

None declared.

## Data Availability

Sequence reads are available on GenBank under the accession code PRJNA472842 (Duranton et al., 2018).
